# Stages of change: A qualitative study on the implementation of a perinatal audit programme in South Africa

**DOI:** 10.1186/1472-6963-11-243

**Published:** 2011-09-30

**Authors:** María Belizán, Anne-Marie Bergh, Carolé Cilliers, Robert C Pattinson, Anna Voce

**Affiliations:** 1 MRC Unit for Maternal and Infant Health Care Strategies, University of Pretoria, Private Bag X323, Arcadia 0007, South Africa; 2 School of Social and Government Studies, North West University, Potchefstroom Campus, Building F13, Hoffmann Street, Potchefstroom 2520, South Africa; 3 Department of Public Health Medicine, School of Public Health, George Campbell Building, Science Drive, Howard College, University of KwaZulu-Natal, Durban 4041, South Africa

## Abstract

**Background:**

Audit and feedback is an established strategy for improving maternal, neonatal and child health. The Perinatal Problem Identification Programme (PPIP), implemented in South African public hospitals in the late 1990s, measures perinatal mortality rates and identifies avoidable factors associated with each death. The aim of this study was to elucidate the processes involved in the implementation and sustainability of this programme.

**Methods:**

Clinicians' experiences of the implementation and maintenance of PPIP were explored qualitatively in two workshop sessions. An analytical framework comprising six stages of change, divided into three phases, was used: pre-implementation (create awareness, commit to implementation); implementation (prepare to implement, implement) and institutionalisation (integrate into routine practice, sustain new practices).

**Results:**

Four essential factors emerged as important for the successful implementation and sustainability of an audit system throughout the different stages of change: 1) drivers (agents of change) and team work, 2) clinical outreach visits and supervisory activities, 3) institutional perinatal review and feedback meetings, and 4) communication and networking between health system levels, health care facilities and different role-players.

During the pre-implementation phase high perinatal mortality rates highlighted the problem and indicated the need to implement an audit programme (stage 1). Commitment to implementing the programme was achieved by obtaining buy-in from management, administration and health care practitioners (stage 2).

Preparations in the implementation phase included the procurement and installation of software and training in its use (stage 3). Implementation began with the collection of data, followed by feedback at perinatal review meetings (stage 4).

The institutionalisation phase was reached when the results of the audit were integrated into routine practice (stage 5) and when data collection had been sustained for a longer period (stage 6).

**Conclusion:**

Insights into the factors necessary for the successful implementation and maintenance of an audit programme and the process of change involved may also be transferable to similar low- and middle-income public health settings where the reduction of the neonatal mortality rate is a key objective in reaching Millennium Development Goal 4. A tool for reflecting on the implementation and maintenance of an audit programme is also proposed.

## Background

There are many proven strategies likely to succeed in changing behaviour in health practitioners, thereby improving maternal, newborn and child care or reducing mortality [1]. Audit and feedback is a longstanding clinical practice in this area of care. According to Ronsmans [2], the definition of audit provided by Crombie and colleagues in 1997 is the most widely used: "The systematic and critical analysis of the quality of medical care, including procedures used for diagnosis and treatment, the use of resources, and the resulting outcome and quality of life for the patient." Audit is therefore considered important for identifying deficiencies in care by examining a few cases, which could reveal major health service problems. It is further assumed that if solutions to these problems were recommended, actions would be implemented to address the deficiencies [3]. From their systematic review Jamtvedt *et al. *[4] concluded that audit and feedback could be effective in improving professional practice, although the effects were mostly small to moderate. In the case of a low baseline adherence to recommended practices and more intensive feedback, the relative effectiveness could be greater. In the area of perinatal mortality, a systematic review of critical incident review in perinatal and maternal mortality found no randomised trials in this field [5]. A meta-analysis of effects associated with the introduction of perinatal audits in middle- and low-income countries did however demonstrate a 30% reduction in mortality [3].

The Perinatal Problem Identification Programme (PPIP) was developed in the 1990s by the South African Medical Research Council's Research Unit for Maternal and Infant Health Care Strategies (MRC Unit) and is supported by the Department of Health. Various sponsors have contributed to the project over the years, including the South African Medical Research Council, Health Systems Trust, Save the Children and the Centers for Disease Control and Prevention (CDC). The programme was intended to introduce health professionals to an audit tool for the improvement of the quality of perinatal care in the public health care sector. Private hospitals were not targeted *per se *for using PPIP, as they cater for less than 10% of births in South Africa. PPIP has also not been designed as a national monitoring tool - that is taken care of by the district health information system (DHIS). Apart from determining perinatal mortality rates (PNMRs), PPIP also identifies the avoidable factors associated with each death. The assumption is that the identification of these factors will lead to recommendations for improvement.

The MRC Unit initially invited clinician volunteers in public sector hospitals to join and apply the programme in their own institutions. Currently 326 of the 664 public health facilities (49%) conducting deliveries in South Africa are registered on the PPIP database coordinated by the MRC Unit. Training sessions and updates in the use of the PPIP software is provided in different provinces on a regular basis when there is a demand. For the 2008-9 analysis, the 275 facilities (41%) that submitted PPIP data were responsible for 52% of all births in the public sector. These institutions included 10 out of 15 tertiary hospitals, 50 out of 65 regional hospitals, 170 out of 257 district hospitals and 45 out of 327 community health centres/midwife obstetric units (MOUs). Data is not collected from private institutions using the programme. PPIP has also been introduced to countries like Namibia, Zimbabwe, Lesotho, Swaziland and Maldives (personal observations) and is used in some hospitals in Bangladesh (personal communication, Louise T Day).

As the use of PPIP expanded, a need arose for coordinators to manage the process and to collate provincial data. In some provinces coordinators are now officially assigned to run PPIP in the province, collate data from the individual sites and report back on a regular basis. PPIP produces regular *Saving Babies *perinatal survey reports on findings [6]. In the pilot stages of the implementation of PPIP, two studies that showed a significant reduction in perinatal mortality demonstrated the power of audit [7-9]. PPIP has been used to create baseline data and to monitor subsequent change.

Although the literature is clear on the importance of audit, little information is available on the processes involved in the implementation of audit systems in health care settings in middle- and low-income countries and on conditions that facilitate or hamper the implementation and sustainability of such systems. Our study presents the outcomes of an attempt to understand the processes involved in initiating and implementing an audit programme, as well as factors contributing to the sustainability of the programme over a period of time.

## Methods

A qualitative research approach was adopted to explore clinicians' experiences of the initiation, implementation and maintenance of PPIP. The main point of data collection was a workshop organised for the Synergy Group, a group of administrators of PPIP and the Child Problem Identification Programme (Child PIP) (mostly doctors, midwives and nurses) working in the 35 government sites with serial audit data for PPIP for a period of at least five years and the 23 sites with serial data for Child PIP for at least three to four years. The workshop was attended by 48 participants, which included 17 PPIP and 12 Child PIP clinicians, seven regional or provincial coordinators (some responsible for both PPIP and Child PIP) and a number of 'outsiders' with experience in implementing and sustaining other types of programmes. Our analysis focused on the experiences of the PPIP clinicians and coordinators.

The study was approved by the Research Ethics Committee of the Faculty of Health Sciences of the University of Pretoria. Each workshop participant signed informed consent for the data to be used anonymously and the managers of the participants' institutions signed permission for their attendance of the workshop.

Two workshop breakaway sessions were devoted to the implementation and maintenance of audit programmes. The participants in each session were divided into four groups of 10 to 11 participants each. All participants, except for four, were doctors, midwives or nurses involved in activities related to PPIP and/or Child PIP. These groups will be referenced in the direct quotations as [G1] to [G4]. Data from Session 1 (Implementing an audit system) and Session 2 (Sustaining audit) formed the basis of the analysis for this paper. For Session 1 [S1] participants were divided according to the level of care: two groups of district hospital (level 1) representatives, one group of regional (level 2) hospital representatives, and one group consisting of a mixture of regional and provincial (level 3) hospital representatives. In Session 2 [S2] participants were mixed, so as to include representatives of different levels of care, different professions and different provinces in each group.

Group facilitators received detailed guidelines on issues to discuss. Each group had an observer who acted as a recorder (scribe) and who made detailed field notes of the discussion (Data source 1 [DS1]). Main ideas were recorded on flipcharts developed by the group participants (Data source 2 [DS2]). Each participant had a note sheet to complete at the end of each session on which he or she indicated the three main points that had most struck him/her in that session (Data source 3 [DS3]). In the plenary feedback session each group report was followed by a general discussion. Three independent observers also took field notes of these proceedings (Data source 4 [DS4]). The four data sources served as a form of data triangulation.

Two researchers (MB & CC) did the initial thematic analysis. Their preliminary findings were discussed by eight members of the Synergy Group and further input was provided. A third researcher (A-MB) then carried out a further analysis to identify relationships between themes. This was followed by a round of joint meetings of the researchers to further refine the analysis.

### Analytical framework

**Figure 1:**
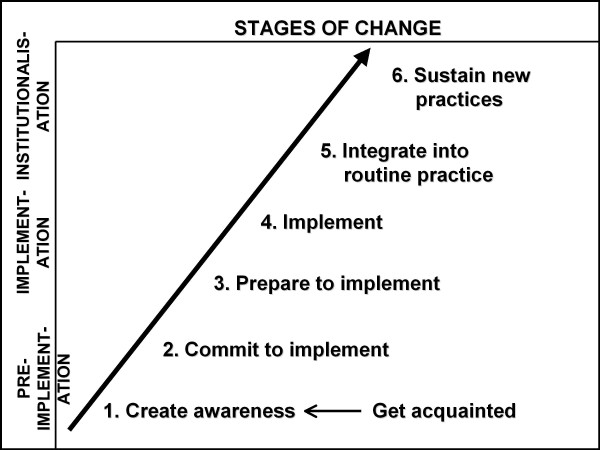
**Model for understanding the stages of change in the implementation and sustainability of audit**.

The analysis started out with a grounded theory approach, but it soon became clear that a stages-of-change conceptual framework would be useful for the further analysis of the data and the description of findings. This framework was derived from an organisational change model initially developed for measuring progress in the implementation of kangaroo mother care as a new health care intervention [10]. This model is depicted in Figure 1. It has three phases: pre-implementation, implementation and institutionalisation. Each phase has two 'stages' or 'steps', beginning with awareness of the importance of audit. This is followed by stage 2, the adoption of the concept of perinatal audit and a commitment to implement the programme. The implementation phase begins with preparation for implementation (securing the human, physical or financial resources) (stage 3). Implementation (stage 4) starts when the first audit evidence is produced. The institutionalisation phase entails the integration of audit into existing practice so that it becomes routine, including regular discussion of findings at perinatal review meetings and the implementation of recommendations (stage 5). The final stage refers to the ability to sustain audit, which is demonstrated by the availability of serial data over a period of at least three to five years.

Our model makes provision for understanding change, a process that needs to take place within the context of a group or team working together to make change happen. It is also compatible with quality improvement cycle models [11,12] and many of the theories encompassing stages of change and/or processes of change and behaviour change models [13-18].

## Results

It is widely advocated in the literature that the implementation of programmes such as PPIP should be thought of as a process and not a once-off event [19]. Thinking of implementation and sustainability as a process helps to highlight the fact that different tasks, events or people assume more prominent or lesser roles, depending on the stage of the process. Implementation as a process also acknowledges that change in attitudes, skills and behaviour is gradual and that ongoing support is crucial.

For each of the six stages certain activities have to take place and certain factors have to be present. These factors serve as conditions that could either hamper or facilitate the implementation actions of a health care facility or district.

### Essential factors for successful implementation of an audit system

From the data we identified four themes that were important in successfully implementing and sustaining PPIP: drivers and teams, outreach and supervision, perinatal review meetings, and communication and networking. They are discussed below.

#### Drivers and teams

Workshop participants agreed that drivers or agents of change were a key facilitating factor in the change process of implementing and sustaining audit. The driver was considered to be an "*interested person who is [the] driver to roll out PPIP & stay[s] motivated to make improvements" *[DS3-S1-GC]. PPIP drivers at facility level were identified in different ways. In some instances the regional or provincial coordinator or the hospital manager allocated the task to a particular individual or group. In other cases a naturally interested person or persons took ownership of the programme. These drivers were described by all groups of participants as "*enthusiastic"*, "*passionate"*, "*committed"*, "*responsible" *and *"motivated"*. They were managers or clinicians (doctors, nurses, midwives) who took responsibility for installing the software and organising the data entry. One participant noted: "*Midwives/Nurses are often appropriate drivers at institutional level" *[DS3-S1-GC].

Participants also acknowledged the importance of team work - "*We need more than a driver for sustainability; we need a team" *[DS1-S2-GC]. As teamwork involved a group of people, this diminished the risk of the programme's failing when a single driver was unable to continue with PPIP activities. Participants also referred to the importance of having management on board to establish an audit team: "*Team work requires buy in from top to down" *[DS3-S1-GB].

Drivers and teams operated at different levels, ranging from the primary health care level, through to the hospital, the district and the province. In addition to the need to have a key person and team driving the process at facility level with the support of senior management, it was also clear that the broader success of the implementation of the programme depended on committed drivers and teams at higher levels, which in South Africa included Maternal, Child and Women's Health (MCWH) role-players at sub-district, district and provincial level - *"Having a key person in the facility, a driver, makes it successful. But for wider expansion, it seems to require MCWH management" *[DS4-S1-GC]. One participant commented as follows on the note sheet, combining the issue of a driver, the team and support from the health system: "*Leadership - person-based & driven; with development of team + support of that PPIP person by regional Coordinator" *[DS3-S1-GA].

#### Outreach and supervision

Workshop participants used the term 'clinical outreach' to refer to the efforts of health professionals to improve quality of care in the health care facilities in a defined area such as a health district, region or province. It is supposed to be an activity involving mutual engagement, which is often educational in nature, such as teaching a specific skill or finding solutions for problems related to the health system (e.g. managerial or resource issues). In our study an outreach person (or persons) acting as a driver was reported as playing an important role in introducing PPIP to a health care facility and in supporting the process - "*Support from outreach clinic person essential" *[DS3-S2-GB]. Outreach persons also oversaw the regular collection, capturing and analysis of data. As supervisors "*with a dedicated role of outreach" *[DS1-S1-GC], they were in a position to identify future in-facility drivers to take the process forward. One of the groups summarised the importance of outreach and supervision on their flipchart notes as follows:

*"It is important to have an 'over-see-er' at facility level and DOH [Department of Health] Outreach person provides support and training" *[DS1-S1-GC]

Outreach programmes and the role of supervision were structured differently in different provinces. Some provinces had no formal outreach programme for the implementation of PPIP; in others the provincial or regional MCWH coordinator or another appointed provincial official fulfilled this support function. Community paediatricians or obstetricians were also involved in certain areas. With regard to the role of coordinators in sustaining audit, the session notes of one group referred to a specific coordinator, with one participant commenting: *"In our province, she [the PPIP coordinator] doesn't allow people to be unsustainable. She checks up. Without her, half of the sites will vanish" *[DS1-S2-GA].

#### Perinatal review meetings

Institutional review and feedback meetings were a pivotal point throughout the implementation and maintenance process of audit programmes - "*Meetings are for identifying the problems and try to avoid them in future" *[DS3-S1-GC]. Workshop participants felt that using existing meetings to report on audit findings and to make recommendations was more efficient than creating separate structures. Three different types of meetings with different functions were mentioned:

• Daily or weekly meetings in the ward, unit or department where deaths were analysed as they occurred.

• Monthly perinatal review meetings at hospital level were very important and provided a means of encouraging continuous data collection - *"Keep on with perinatal meeting → keep PPIP on to collect the data needed" *[DS3-S1-G3].

• Annual meetings held at regional, provincial or national level to report on aggregated death rates and causes of death - "*Yearly PPIP meetings - there is an aim in the end" *[DS1-S2-GA].

Preparation before review meetings was considered vital. Specifically, some sort of agenda had to be made available and presentations prepared. A perinatal audit checklist was considered a useful tool for conducting meetings. Participants emphasised that meetings should be conducted in a positive spirit and should demonstrate the use, benefits and validity of the programme. Resistance could be minimised by helping health workers understand that audit was not a punitive system and that "*meetings should not be a blaming exercise (this often happens)" *[DS1-S1-GD], but should rather be considered a "*learning curve" *[DS4-S2-GB]. Individuals should be confronted in a confidential one-on-one situation before a meeting - "*Don't make people look stupid in front of their colleagues" *[DS1-S2-GC].

Two important outcomes of meetings identified in the workshop were the keeping of minutes (with decisions and actions clearly marked and with the names of the people who were to follow up on the actions) and the compilation of regular reports. Quarterly reports feeding into the annual institutional report were advised. Further training of staff was also mentioned as an outcome of some review meetings.

#### Communication and networking

A further recommendation by workshop participants regarding the implementation and maintenance of any audit system was that the complexity of the South African public health system should be taken into account. Participants identified networking and communication between different levels in the health system, different sites and different role-players as an important facilitating factor in the establishment of PPIP - "*Networking and communication is necessary right through Provincial, Regional, sub-districts, all levels of care" *[DS3-S2-GA].

*"There was support from regional office ... It is very important to network in level 1 and level 2 facilities. Working together and communicate and support each other. Networking, communication, buy-in: management - from the top, then downwards." *[DS1-S1-GA]

In addition to the perinatal review meetings, which are described above as the backbone of the continuation of audit and the completion of the audit cycle through the implementation of recommendations, a well-defined pathway for the flow of data and findings from the primary health care level up to the provincial or national level was also considered essential. MCWH coordinators and district health information system officers were regarded as key role-players in this process. Furthermore, the provision of feedback to the lower or other appropriate levels of care in an institution was considered a further responsibility to be built into any perinatal audit system. It was considered important to include the key role-players at primary health care clinics and MOUs in hospital perinatal review meetings so as to address modifiable or avoidable factors at these levels through joint recommendations. Similarly, it was recommended that the district hospital be part of any outreach programme coordinated by a regional or provincial hospital and the district or sub-district office. Strong communication and networking that require timely submission of data can give health workers a sense of accountability and a sense of doing something worthwhile.

### Stages in the implementation and maintenance of the audit programme

Although the four themes discussed above stood out as key to the success of implementing and sustaining PPIP, they were interrelated, with no neat boundaries between them, and they informed each other. These themes were important throughout the different stages of change, albeit in slightly different ways. To illustrate these complexities and provide a deeper understanding of the interrelation between the four themes, we discuss the responses of the participants according to the analytical stage-of-change framework introduced in the Methods section.

#### Pre-implementation phase

This phase refers to the ways in which hospital clinicians learned of the existence of PPIP, and how they came to understand the benefits of having such a programme to monitor the quality of perinatal care and decided to start PPIP.

##### Stage 1 - Create awareness

In the first stage of change an individual or a team has become aware of a problem, for example high perinatal mortality rates. At this point some hospitals have already perceived the need to collect data on mortality rates and causes - *"We understood services had to be improved" *[DS1-S1-GD]. Some hospitals even had their own system of audit linked to their regular mortality review meetings, as explained by a clinician from a district hospital:

*"In 1999 we just started collecting data. ... I felt there was something wrong. We needed to get a handle on it. Why are babies dying? We didn't get far ... Then we were invited to a provincial PPIP meeting in 2002 or 2003. We got the software ourselves and we were excited about the tool ... Spreadsheets helped. PPIP really helped to give some analysis." *[DS1-S1-GA]

In hospitals where no audit system was in place a growing need was felt for a programme to address the improvement in quality of care and the reduction of perinatal mortality. This led to awareness of the existence of PPIP. The programme was sometimes discovered at a conference or through the visit of a consultant:

*"Thirteen years ago, Prof[essor] A came and told us, 'You and Hospital X must start it'. We heard what it was all about and we went with it." *[DS1-S1-GC]

Most participants indicated that PPIP had been introduced by an outreach person who identified drivers and promoted the programme at other regular meetings at the hospital. A provincial coordinator commented as follows:

*"We phoned all the hospitals and said, 'We have to start it [PPIP].' I offered to come to train everyone on the programme and the hospital manager decided in the labour or paeds ward who will carry on with the audits after training." *[DS1-S1-GC]

##### Stage 2 - Commit to implementing audit

As more health practitioners, administrators and management *"from top to bottom" *[DS3-S1-GA] bought in on the concept of a perinatal quality improvement programme, there was more commitment to implementing PPIP.

*"What was very important was the buy-in from everyone, to inform everyone what it [PPIP] was about. We informed our [regional] director and got his buy-in. Then [you] inform management at each place. Also, to inform management in each sub-district and they must identify a responsible person to do PPIP, et cetera." *[DS1-S1-GA]

A critical mass receptive to the potential benefits of using PPIP facilitated commitment to implementation at hospital level. The adoption of the idea was normally formalised as a commitment by senior management, a clinician, or a group of clinicians and was facilitated by using existing meetings and communication channels to get people on board and institute the programme.

#### Implementation phase

This phase refers to the ways in which PPIP was implemented. It entails the practical aspects that had to be addressed to get PPIP up and running. These include all the preparations needed, as well as the collection, processing, analysis and dissemination of the initial data, findings and recommendations.

##### Stage 3 - Prepare to implement an audit programme

Workshop participants discussed some of the preparations needed before PPIP could be fully implemented. Two major actions at this stage were the acquisition and installation of the necessary software and the training of designated people to collect and enter the data - *"You don't need permission to start PPIP. You just need a driver, the software and some training" *[DS4-S1-GD]. The availability of computers facilitated or hampered the ultimate implementation of PPIP. In some facilities there was an *"issue of departmental policy" *[DS1-S1-GC] where drivers of the implementation process had found it difficult to convince the health informatics officials of the value of the PPIP programme and that it was not a *"Mickey Mouse programme" *[DS1-S1-GC]. The designated or self-appointed institutional driver was normally responsible for setting up the programme and organising the system of data entry and feedback.

Because data capturers need to understand the requirements of data entry, the selection of the right person(s) with a particular degree of computer literacy or people who could be trained was an important step. In some instances the driver also acted as data capturer. Getting the facility information officer (FIO) on board to provide continuous support was viewed as a further step towards smoother implementation in some hospitals, but an obstacle in others, where the FIO did not understand the purpose of PPIP, the importance of accurate data entry and the way PPIP data should be interpreted.

Participants believed that where there had been positive support and encouragement from management the process ran more smoothly. This was also the time when people really started taking ownership of the process.

##### Stage 4 - Implement audit

Implementation began with the collection of the first data and reporting on the findings at perinatal review meetings. Information technology featured strongly at this stage. Some facilities regarded the outreach person and/or provincial coordinator as a pivotal support person who would help to promote change and ensure good participation by senior managers, so that they stayed well informed and supportive. Feedback and review meetings were a vital link between the preparation stage and finally making the audit work. During this stage the process of buy-in and taking ownership continued as the teething problems were sorted out. The demonstration of the use and validity of the audit tool served as a facilitator - *"It worked well because it was central to our goals" *[DS1-S1-GC].

Additional facilitators of implementation included district requirements for information and feedback, a good communication system that ensured regular feedback and a high sense of accountability. Continuing education and refresher sessions on the use of the software for data entry helped to sustain the process. The practice of making back-ups and analysing the data was also followed. An important lesson for one workshop group was immediate and regular data entry in order to obtain accurate data for high-quality analyses - *"Inaccurate if left till late" *[DS2-S1-GB]. PPIP's user-friendliness was a further incentive to enter data immediately. Not understanding the importance of making regular electronic back-ups led to the loss of data in a few instances - *"I have copies in several places" *[DS1-S2-GA]. In most institutions one computer had multiple users and *"IT sees this software, doesn't know it and deletes it" *[DS1-S2-GC].

#### Institutionalisation phase

PPIP achieved the institutionalisation phase when the process of collecting, analysing and distributing the data and using the results was integrated into routine practice and had been sustained for some time - *"Starting it is the easy part; keeping it going is the challenge" *[DS1-S1-GC].

##### Stage 5 - Integrate audit into routine practice

In the institutionalisation phase, the programme or practice becomes part of institutional routine and relevant activities are performed by everyone required to do so. Stage 5 was the point at which PPIP became part of the routine activities of the health care facility and at which the team began to get a clear sense of how they could use the audit findings. The driver was in a position to lead the audit process - *"Whoever is in charge - it must be a formal responsibility" *[DS1-S2-GA]. Although clinicians said that institutionalisation was facilitated where audit duties were included in job descriptions and where staff members were allocated work time for this, it was also stated that:

*"It's critically important that people don't hide behind a busy schedule to avoid doing audit. It's not an extra; it's as crucial as filling in your clinical notes. If you can get people to run with the idea that it's integral, you don't need to beat them to do it." *[DS1-S2-GC]

Outreach people also played an important role in assisting facilities to interpret their findings and to suggest possible improvements. Another key point was *"IT infrastructure back-up" *[DS2-S2-GC].

Some hospitals were of the view that if the use of a particular audit programme could be implemented as national policy then the chances of institutionalisation would be greater. Participants proposed that this institutionalisation should include some form of accreditation and a system of recognising good practice.

##### Stage 6 - Sustain audit

The audit sites represented at the first workshop in our study were those with serial data, which means that they had all reached the stage of being able to sustain the running of an audit programme. Furthermore, there should be evidence that the feedback is followed by recommendations for improvement. Ultimately there should be evidence of the implementation of the recommendations and a demonstration of impact on quality of care and, if applicable, trends in perinatal mortality rates. Different sites used different modalities to sustain audit - *"different people, different places, different plans" *[DS2-S2-GD]. Although there were no 'recipes' or simple guidelines on how to sustain audit, workshop participants were unanimous about the importance of ownership that *"should be very deep" *[DS1-S2-GA].

Regular perinatal review meetings, with *"decent preparation" *[DS1-S2-GC] are crucial in reaching the point of sustainability. At the annual provincial and/or national meetings individual hospitals or provinces were required to present the data from their institution or province, providing further impetus for continuing with PPIP. Meetings such as these were also used to demonstrate the benefits of using PPIP.

Previously discussed factors that enhanced the possibility of sustaining PPIP were outreach visits and staff stability, with at least one person taking responsibility for PPIP in the health facility on a permanent basis. The emphasis was furthermore on team work, as having PPIP in the hands of a single individual without back-up could be a threat to sustainability - *"Have more than one interested person to run the programme" *[DS3-S2-GC]. *"Succession planning" *[DS3-S2-GA] was also mentioned as a prerequisite for sustainability.

## Discussion

This study aimed to make visible the processes involved in the implementation and maintenance of the PPIP audit programme used in South African public hospitals and to explore the factors contributing to the successful implementation and sustainability of PPIP. A stages-of-change conceptual framework that encompasses an understanding of behavioural change was used for the analysis of the data and the description of the findings was developed inductively from the data. The fact that only 35 institutions had managed to collect serial data for five years or more illustrates the difficulty of sustaining audit and feedback and points towards the need for more research into health systems weaknesses and ways of strengthening audit practices.

Change is thought of as a complex process in which different factors act as barriers or facilitators [20]. No single factor is responsible for bringing about change. Change takes place in a context in which several different factors, in interaction with each other, contribute to the successful implementation and maintenance of an audit programme [21,22] - "multiple and often unpredictable interactions arising in particular contexts and settings ... determine the success or failure of implementing changes" [13].

Armenakis and Bedeian distinguish between content, context, process and outcomes of change in their review of the theory and practice of organisational change theory and practice [23]. When their typology is applied to the implementation of an audit programme the content is the audit programme itself. The context is the hospital or MOU where the programme has to be installed and used. The process could be equated to the six stages in the change process, from being aware of the problem and the existence of the audit programme to the sustainable use of the programme. The outcomes of the change would be an audit system that is sustainable and able to provide regular results.

The four main themes or factors we identified for the successful implementation and maintenance of audit correspond to findings from other studies. With regard to *drivers *or change agents, Pattinson and Bergh distinguish between "enablers" and "doers" in the context of implementing recommendations following confidential enquiries into maternal deaths [20]. Applied to perinatal audits, the doers would be the clinicians responsible for service delivery, for implementing the audit system and ensuring that the necessary recommendations to complete the audit cycle are implemented to change practice and improve quality of care. Enthusiastic doers are more likely to report on improvements in perinatal mortality in their institutions than those who are less involved. This may explain why the three studies done in South Africa on the reduction of perinatal mortality after the introduction of PPIP all found a significant decrease [7-9,24]. This is contrary to the findings of the systematic review by Jamtvedt and colleagues [4].

Change agents acting as enablers would include IT support personnel, provincial coordinators, managers and outreach clinicians who would, for example, look after procurement, staff development (training) and staff allocation (e.g. giving staff time to perform their audit duties adequately). For change agents to operate successfully, there must be appropriate formal and informal *communication *channels (with proper information flow) [25], some form of clinical or specialist *outreach *[26,27] and other networking opportunities throughout the health system [20].

Even if an audit programme has been implemented and sustained, this does not guarantee the implementation of recommendations emanating from the regular audit and feedback. Changing one aspect, such as introducing an audit programme, means many other aspects would also change [20]. Conducting audit and giving feedback at morbidity and mortality meetings and other educational and review meetings still does not mean that quality of care will improve automatically. Self-correction of behaviour, the active involvement of health professionals in the change process and the allocation of formal responsibilities for implementing and monitoring recommendations have been put forward as possible ways of effecting change [20]. When the implementation of recommendations following on feedback is continuously evident, the audit cycle has been completed and sustainable audit achieved. This would be reflected in the impact indicators set by the facility, such as mortality rates or other quality of care indicators, a matter which was beyond the scope of this study. For measuring the completion of the audit cycle, a similar study of the process of stages of change to the one described in this paper could be embarked upon to explore the achievement of change in practice.

The implementation of PPIP, introduced as a voluntary audit programme, initially followed a grassroots trajectory in implementation by institutions wishing to make a difference by voluntarily adopting PPIP. Each facility that accomplished implementation and maintenance of PPIP achieved success by using their own resources and knowledge or experience. As awareness of the benefits of PPIP increased by word of mouth and reports at meetings and conferences, some provinces identified a need for across-the-board implementation and directives started coming 'from above'. The ways or strategies the facilities used to implement PPIP were not based on any theories of behavioural change nor on evidence-based directives received from an authority in the health system. However, hospitals in our study used very similar strategies, possibly because they were part of the public health system and the networking activities that followed the introduction of PPIP. Their strategies also resonate with those described in other studies on the improvement of quality of care, such as educational meetings, the influence of opinion leaders or outreach visits [1].

Viewing the implementation and maintenance of an audit programme as a process, as described in this paper, could be helpful in ensuring the success of future efforts to implement audit programmes like PPIP. Table 1 provides a list of questions that could be used as a tool for implementers or users of audit programmes before they start implementing such a programme or as a reflection tool to help them assess progress. The question topics could be considered as issues that need to be addressed at each stage of implementation.

**Table 1:** Tool for reflecting on the implementation and maintenance of an audit programme

STAGE	QUESTIONS TO CONSIDER
**Stage 1**: **Create awareness** • of problem (e.g. high PNMR) • that something must be done • of audit programmes that could be used	• What is the level of awareness of staff about the problem? • How can we create or improve a general awareness of the importance of audit among staff? • Do we have review meetings (e.g. perinatal review meetings) on a regular basis? ○ If no, how could we organise and institute these meetings? ○ If yes, how could we use the existing meetings to create better awareness of the importance of audit? • Should a specific person at our facility be designated to find out about the programme (e.g. PPIP)? • Could a representative of an institution using the audit programme or the owners of the programme (e.g. PPIP) be invited to come and tell staff about it? • Are other facilities in our area/district using audit programmes? ○ If no, what role can our facility play in reaching out to create awareness of the problem and the importance of audit?

**Stage 2**: **Commit to implement audit** • More people commit to implement programme	• Can existing review meetings be used to facilitate commitment from more people? ○ How could we use this? ○ What needs to be done? • Can existing communication channels be used to facilitate commitment from more people? ○ How could we use this? ○ What needs to be done? • Are there specific persons whose commitment is required who need to be approached? ○ People at management level? Who? How? ○ IT officials? Who? How? ○ Clinicians? Who? How? ○ Government officials? Who? How? ○ Other? Who? How? • What steps are needed to get the use of the programme approved in principle? • What kinds of commitment are needed? (E.g. Who? What? Where? When? How?) ○ Written? ○ Verbal?

**Stage 3**: **Prepare to implement** • Practical aspects to get the programme up and running	• What do we expect of the driver(s) of the process? • How will we identify the driver(s)? Who should be considered? • What are the financial outlays for getting the programmes and necessary equipment? ○ Must the audit programme be purchased or is it free? ○ What will orientation and training in the use of the programme cost? ○ Do we have a dedicated computer for capturing audit data? - Should more computers be made available? • How do we get budget approval for the purchase of the programme and other equipment (if applicable)? • What kind of support do we have from senior management on the practical aspects of the audit implementation process? ○ In what areas do we need additional support? ○ What else should we do to secure the necessary support? • Has the software for the audit programme been installed? • Who will be responsible for data entry? ○ Has this duty been negotiated with the assigned data capturer(s)? - How well have they bought into the idea? ○ What kind of computer training do they need? ○ What kind of training do they need in the use of the software? - How will that be organised? ○ What kind of support or incentives should be provided to the data capturers to make them aware of the value of their work? • What kind of quality control will be exercised on the captured data? • Who has been trained in interpreting the data and generating reports? ○ If no one, what should be done about it? • Are all the responsible clinicians trained in the use of the computer hardware? • Are all the responsible clinicians trained in the use of the software? • How are facility information officers or IT officials involved in the establishment of the system? ○ How informed are they? ○ What kind of support will they be able to provide? • What kind of support do we need from our outreach clinicians and other health department officials in our preparation for implementation? ○ How could these person(s) assist us in keeping the process on track or speeding it up?

**Stage 4**: **Implement audit** • Collection of the first data • Analysis of the results • Dissemination of results	• Have we started collecting data? • How do we keep back-ups of data files? ○ Who is responsible for making back-ups? ○ How often are back-ups made? ○ How do we check that this is done regularly? • Are all persons sharing the computer(s) aware of the programme? (To avoid unintentional deleting of files or programmes) • How can accuracy and regular data entry be improved? ○ Are data always entered immediately? ○ Should refresher sessions on the use of the software be provided? • How is/are the driver(s) of the implementation process coping with the tasks? ○ What kind of additional support do they need? • How is feedback of results given at the review meetings? ○ How can we improve on this feedback? • How good is the attendance of review meetings? ○ How can it be improved? ○ Are attendance registers kept for review meetings? - If yes, how does it help us in what we are doing? - How do we use the information to ensure that all relevant role-players are invited regularly and/or have the opportunity to attend? • Is the usefulness of the audit programme (e.g. PPIP) evident at meetings? ○ If yes, how? ○ How can this be improved? • Are minutes taken at review meetings? ○ If no, how will we get this process going? ○ If yes, how can we improve on what we are doing? - How are the minutes used? - How are recommendations noted in the minutes? - Are timelines and the allocation of specific persons included in the recommendations? • How are the recommendations incorporated into in-service training? • How does the use of the results contribute to accountable behaviour of all relevant role-players? • What kind of support do we need from our outreach clinicians and other health department officials in the implementation process? ○ How could these person(s) assist us in keeping the process on track or speeding it up? • How can communication at different levels be improved? ○ Between providers at the coal face? ○ Between providers and their immediate managers? ○ Between managers and senior management? ○ Between the facility and the other levels of the health system? ○ Other?

**Stage 5**: **Integrate audit into routine practice** • Data collection • Analysis of the results • Dissemination of results • Use of findings ↓ Routine practice	• Are audit duties written into the job descriptions of relevant staff members? • Is feedback on audit results regularly provided to all relevant service providers in the institution? ○ How can we improve on this? • Is feedback on audit results regularly provided to senior management in the institution? ○ How can we improve on this? • Is feedback on audit results regularly provided to higher levels in the health system (e.g. district, regional or provincial managers)? ○ How can we improve on this? • How are the audit data interpreted? ○ How can we improve on this? • What kinds of recommendations and improvements are suggested following the interpretation of data? ○ How can we improve this? • How are we faring with the execution of the recommendations? ○ What are we doing well? ○ How can we improve on the things we are not doing so well? • Does our facility need (more) visits from an outreach person or team to assist us with the institutionalisation of the audit programme?

**Stage 6:** **Sustain audit** • Data collection • Analysis of the results • Dissemination of results • Use of findings ↓ Sustained over a longer period of time	• How can the regular review meetings be improved and used more effectively? • How often and to whom is feedback given? ○ Monthly? To whom? ○ Quarterly? To whom? ○ Six-monthly? To whom? ○ Annually? To whom? • What are the gaps in our feedback procedures? • How can the feedback to service providers and senior management in the facility be improved? ○ How can engagement in the audit process, the use of the findings and the application of recommendations be improved? • How can the feedback to the higher levels of reporting be improved (e.g. district or provincial levels)? ○ How can involvement from these levels be improved? • Who is responsible for keeping the audit system together? ○ One person? ○ A team? • Who is leading the audit? ○ Who takes responsibility when the leader(s) is/are not there? ○ What kind of succession plan do we have? • How do staffing issues such as rotations and turnovers influence the audit activities? ○ How can staff stability be improved? • What is our facility's responsibility in reaching out to another facility or facilities to introduce and establish an audit programme (e.g. PPIP)?

## Conclusions

The findings of our study contribute to knowledge of the processes involved in implementing and sustaining quality of care and mortality audit-and-feedback programmes. They also highlight the notion of implementation and sustainability of an audit programme as a process that can be conceptualised as stages of change, each with a different allocation of roles, tasks and events.

Conditions that appear to be essential for the implementation and maintenance of audit are the continuous presence of drivers and teams, outreach and supervision, regular morbidity and mortality meetings, and communication and networking. Other facilitators include information and feedback from a higher level in the health system, initial training and regular updates in the use of the software, and a sense of accountability and motivation in those responsible for the immediate and accurate entry and analysis of data. Being prepared for potential facilitators and barriers could guide future tailored interventions for the implementation of audit programmes.

Our study was intended to get a better understanding of how a perinatal mortality audit system was introduced and sustained, despite the complexities of the South African public health system [28,29]. The findings may therefore not be generalisable in their application in other countries. However, insights into the factors necessary for the successful implementation and maintenance of an audit programme and the process of change involved may also be transferable to similar low- and middle-income public health settings where the reduction of the neonatal mortality rate is one of the key objectives on the road to reaching Millennium Development Goal 4. The tool developed for monitoring or reflecting on progress with the implementation of an audit programme has potential for transfer and adaptation for use beyond maternal and child health settings.

## Competing interests

The authors declare that they have no competing interests.

## Authors' contributions

A-MB and RCP conceptualised the design with input from other authors. All authors participated as facilitators or scribes in the main workshop where data was collected. MB, CC and A-MB did the initial data analysis. All authors participated in the interpretation of findings and the drafting and finalisation of the manuscript. They all read and approved the final manuscript.

## Pre-publication history

The pre-publication history for this paper can be accessed here:

http://www.biomedcentral.com/1472-6963/11/243/prepub
